# Advances in the Diagnosis and Management of Congenital Heart Disease in Children

**DOI:** 10.3390/children9071056

**Published:** 2022-07-15

**Authors:** P. Syamasundar Rao, Arpit Agarwal

**Affiliations:** 1 Children’s Heart Institute, McGovern Medical School, University of Texas at Houston, Memorial Hermann Children’s Hospital, 6410 Fannin Street, Suite #425, Houston, TX 77030, USA; 2 Division of Pediatric Cardiology, The Children’s Hospital of San Antonio, Baylor College of Medicine, San Antonio, TX 78107, USA; arpit.agarwal@bcm.edu

The last five decades have witnessed an inordinate number of advances in the diagnosis and management of congenital heart defects (CHDs), as reviewed elsewhere [1]. These advances include the detection of CHDs using fetal echocardiography; the identification of critical CHD via the pulse oximetry screening of neonates before discharge from the hospital in addition to conventional methods of CHD identification by routine history, physical examination, and simple laboratory studies, such as chest X-ray and electrocardiogram; the rapid transportation of the so-identified babies to tertiary care centers equipped to care for these vulnerable infants; accessibility to very sensitive noninvasive diagnostic techniques, namely, echocardiography and Doppler, three-dimensional (3D) echocardiography, magnetic resonance imaging (MRI), and computed tomography (CT); the availability and utilization of three-dimensional (3D) visualization technologies, including 3D printing, virtual reality, and augmented reality for surgical pre-planning; the introduction of percutaneous, catheter-based methodologies to address CHDs; developments in pediatric cardiac anesthesia both for percutaneous and surgical interventions; the enablement of intricate surgical techniques to provide corrective treatment to patients with complex CHDs with an alternative of successful palliation or heart transplantation; effective postoperative management; and diligent post-intervention follow-up. These advances have caused positive results that improve the outcome of babies born with CHDs to the point that there are now more adult subjects with CHD than children. In this Special Issue on “Advances in the Diagnosis and Management of Congenital Heart Disease in Children”, some of these advances will be reviewed.

In the first paper, Dr. Pop from the University of Medicine Pharmacy Sciences and Technology of Tirgu Mures and the Tirgu Mures Emergency Institute for Cardiovascular Diseases and Heart Transplant, Tirgu Mures, Romania, compares different contrast agents (Iomeprol 350, Ioversol 350, Iopromide 370, and Iodixanol 320) used for computed tomographic angiography (CTA) studies in infants referred for aortic arch evaluation [2]. Dr. Pop asserts that CTA studies in babies are difficult secondary to variations in the types of the contrast used and the volume and rate of infusion of the contrast material. She performed a retrospective comparison of 4 different contrast materials in 48 consecutive CTA studies in babies less than one year of age. All CTA studies were undertaken with the same 64-slice scanner and used similar power-injection techniques. The results indicated that Iodixanol 320 achieved nearly 40% less enhancement compared to the other three agents in identifying aortic coarctation and aortic arch hypoplasia [2].

In the next paper, Dr. Singh from the Baylor College of Medicine/The Children’s Hospital of San Antonio, San Antonio, TX, USA, reviews “as low as reasonably achievable” (ALARA) in pediatric electrophysiology laboratory [3]. Dr. Singh emphasizes the occurrence of adverse radiation effects (cataracts, skin abnormalities, malignancies, birth defects, and spine and orthopedic injuries) associated with electrophysiologic studies and therapy, particularly in children, and advocates ALARA. He also stresses and supports the abolition of needless exposure to the radiation of patients, physicians, and other laboratory personnel and simultaneously delivering acceptable imaging for making the diagnosis and offering intervention. He concludes that the use of fluoro-less or near-zero fluoroscopy and 3D electro-anatomical mapping techniques are likely to achieve ALARA.

In the third paper, Dr. Divekar and his associates from the University of Texas Southwestern Medical Center/Children’s Medical Center, Dallas, TX, USA, and the Cleveland Clinic Children’s Hospital, Cleveland, OH, USA, review transcatheter device therapy and how to integrate advanced imaging with invasive procedures in the management of CHD [4]. These authors assert that percutaneous device placement is currently being performed as a primary treatment option for several CHDs that have previously been addressed only with heart surgery. The authors also state that new imaging technology is being utilized in the diagnosis, planning, guidance during the procedure itself, and monitoring following device placement. They conclude that advocacy for the patient’s well-being and working in concert with regulatory agencies is germane for the further development of catheter-based device implantation in subjects with CHD.

In the subsequent paper, Dr. Arar and his colleagues at the University of Texas Southwestern Medical Center/Children’s Medical Center, Dallas, TX, USA, review the role of cross-sectional imaging in pediatric interventional cardiac catheterization [5]. This paper has similar attributes as the previous paper but encompasses all pediatric catheter interventional procedures. Both MRI and CT, along with 3D reconstruction, are crucial methodologies and are invaluable tools in pre-procedural evaluation and follow-up assessment and are likely to improve the outcomes of these procedures.

In the next paper, Dr. Betancourt and her associates from The Children’s Hospital of San Antonio/Baylor College of Medicine, San Antonio, TX, USA, review the utility of a three-dimensional printed model in the biventricular repair of complex CHDs [6]. They report a case of heterotaxy syndrome in which a 3D printed prototype allowed them to better comprehend the complex cardiac anatomy and helped in pre-surgical planning and executing a successful biventricular repair. They conclude that the 3D-printed model is likely to advance the comprehension of anatomic complexities and accomplish successful surgical outcomes in complex CHD.

In the final paper of this series, Dr. Sharma and associates from The Children’s Hospital of San Antonio/Baylor College of Medicine, San Antonio, TX, USA, and the Cohen Children’s Medical Center, New York, NY, USA, discuss advances in the prenatal management of fetal cardiac disease [7]. They state that despite the advances in fetal ultrasound studies that have improved the prenatal treatment of the heart, substantial issues remain with regard to how the subjects are selected and how applicable the available therapies are. They review both pharmacological and percutaneous interventions in the fetus and suggest that these treatment modalities have improved remarkably over the years. However, they recommend multi-institutional collaborative initiatives to develop standardized methods for intra-uterine drug therapy and transcatheter interventions and create acceptable guidelines for fetal therapies in an attempt to attain ideal results.

Given the small number (N = 6) of papers included in this Special Issue, not all the advances that happened in the last 50 years could be discussed. Nonetheless, the authors of these papers and we hope that the reviewed material is useful to the reader and help them to provide better care for their patients.
